# Advances in Clinical Outcomes of Endoscopic Lumbar Sympathectomy: Analysis of 494 Consecutive Patients at a Single Institution

**DOI:** 10.3390/jcm14124311

**Published:** 2025-06-17

**Authors:** Duk Hwan Moon, Wongi Woo, Jimin Lee, Sungsoo Lee

**Affiliations:** 1 Department of Thoracic and Cardiovascular Surgery, Gangnam Severance Hospital, Yonsei University College of Medicine, Seoul 06273, Republic of Korea; woopendo@gmail.com (W.W.); rowena7306@gmail.com (J.L.); chestlee@yuhs.ac (S.L.); 2 Department of Internal Medicine, St. Joseph’s Medical Center Stockton, Stockton, CA 95204, USA

**Keywords:** endoscopic lumbar sympathectomy, laser Doppler flowmetry, lumbar sympathetic ganglion block, primary plantar hyperhidrosis, psoas muscle relaxation

## Abstract

**Background/Objectives**: Endoscopic lumbar sympathectomy (ELS) is the definitive treatment for primary plantar hyperhidrosis (PPLH). This study analyzed the mid-term clinical outcomes and technical factors related to ELS. **Methods**: Retrospective reviews of patients who had ELS for PPLH between July 2019 and May 2023 were analyzed. The study period was categorized into three eras based on the timing when laser Doppler flowmetry (LDF) and PMR (psoas muscle relaxation) were applied; period A represented the initial surgical approach, period B included LDF, and period C included LDF and PMR during surgery. The impacts of these techniques on operative and short-term outcomes were assessed. Additionally, risk factor analysis was performed to find relevant factors related to the reappearance of plantar sweating in long-term follow up. As most patients underwent endoscopic thoracic sympathectomy (ETS) as well, risk assessment for compensatory hyperhidrosis was also investigated. **Results**: A total of 474 patients were included, and the numbers of patients by periods were as follows: *n =* 28 in period A, *n =* 198 in period B, and *n =* 248 in period C. Operating times were significantly different, with proportional decreases seen with the introduction of LDF and PMR (*p <* 0.001). In the long-term, reappearance of plantar sweating was noted in 21 patients (4.4%). Risk factors for the reappearance of plantar sweating included an age over 35 years [odds ratio {OR} (95% confidence interval {CI}) 4.57 (1.56–13.40), *p =* 0.006] and a history of lumbar sympathetic ganglion block (LSGB) prior to ELS [OR (95% CI), 269 (29.30–2460), *p <* 0.001]. Of 474 patients, 390 (82.3%) patients underwent both ETS and ELS. Risk factors for compensatory hyperhidrosis were age >25 years [OR (95% CI) 0.60 (0.40–0.90), *p =* 0.014] and concomitant ETS [OR (95% CI) 5.63 (1.88–16.90), *p =* 0.002]. Compensatory hyperhidrosis among patients who only had ELS was less observed (4/24, 16.7%). **Conclusions**: ELS is highly effective in treating plantar hyperhidrosis, and LDF and PMR improved perioperative outcomes. Age over 35 and a prior history of LSGB were found to be related to worse long-term outcomes of ELS. Our findings suggest that ELS with additional LDF and PMR could improve outcomes for patients with PPLH.

## 1. Introduction

Primary plantar hyperhidrosis (PPLH), characterized by excessive sweating of the feet, affects the quality of life and psychological well-being of patients. Persistent sweat accumulation in the feet can result in bacterial growth, leading to malodorous consequences which cause social and interpersonal issues for most patients [1].

Similarly to primary palmar hyperhidrosis (PPH), surgery has been suggested as the ultimate therapeutic option for PPLH compared to nonsurgical interventions [2]. Compared to endoscopic thoracic sympathectomy (ETS), which has become a standardized procedure, endoscopic lumbar sympathectomy (ELS) has emerged as a new technique, and it needs to be studied more with regard to the standardization of the procedures involved and long-term clinical outcomes [3,4,5].

Heterogeneity in terms of ELS outcomes is related to the complexity and variability of the anatomy of lumbar sympathetic chains, which often are a cause for incomplete procedures or inaccurate targeting of lesions [6,7,8,9,10,11,12]. Consequently, ELS is accompanied by an elevated risk of complications, including retroperitoneal bleeding and inadvertent lumbar sympathetic nerve resections [13,14,15]. These complexities raise concerns among surgeons, leading to increased hesitation and avoidance of ELS [16,17].

In this study, we analyzed the effect of integrating laser Doppler flowmetry (LDF) and psoas muscle relaxation (PMR) during ELS to evaluate their impacts on surgical outcomes in ELS. Additionally, risk factor analysis was performed to find any relevant factors which affect clinical outcomes.

## 2. Materials and Methods

### 2.1. Ethical Approval

The study adhered to the principles outlined in the Declaration of Helsinki. Ethical approval for this study was provided by our institutional review board on 26 July 2023 (IRB No. #3-2021-0179). Consent to the study was waived due to its retrospective design.

### 2.2. Patients Selection

This study included patients who underwent ELS between July 2019 and May 2023. The retrospective analysis included a review of electronic medical records to evaluate patient outcomes, including compensatory hyperhidrosis and plantar sweating recurrence. Of 474 patients, 390 (82.3%) underwent concurrent ETS for palmar and/or axillary hyperhidrosis. A thorough examination of patient medical histories, including any prior sympathetic surgery, was conducted as well. However, this study did not include pediatric patients and patients with significant cardiopulmonary comorbidities, who are at high risk for general anesthesia.

### 2.3. Study Definitions and Outcome Assessment

**Figure 1:**
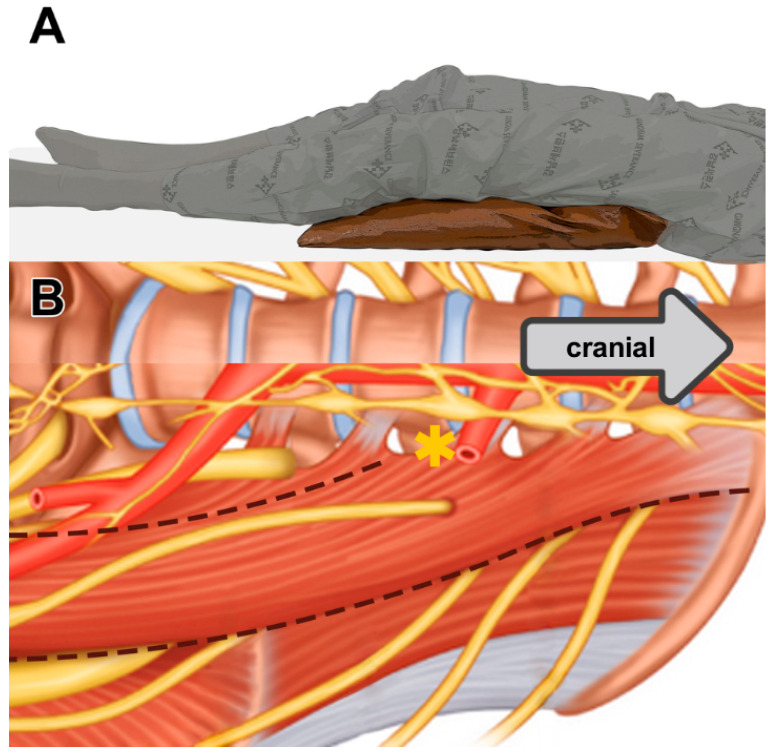
The psoas muscle relaxation (PMR) position leads to widening of the psoas muscle and vertebral space [(*) widen], enabling the exposure of the lumbar sympathetic chain. (**A**) A picture of patient positioning during the procedure. (**B**) Anatomy of psoas muscle and lumbar sympathetic nerve chain.

The study periods were classified based on the introduction of new intraoperative techniques. First, LDF was used to locate the lumbar sympathetic chain by assessing the plantar blood flow; the outcome of this technique was first published in 2022 by our group. Percutaneous blood flow was measured on each side of their soles by noninvasive laser Doppler perfusion monitor probes (Periflux System 5000; Perimed, Stockholm, Sweden) [18]. If the correct lumbar sympathetic chain is grabbed with surgical instruments, there is a significantly acute blood flow drop. This enabled us to target the proper sympathetic chain. Second, PMR was used to improve visibility of lumbar sympathetic chain by relaxing psoas muscle. A pillow was placed behind patient’s knees, and the legs were adequately abducted, creating a comfortable posture. This proper positioning facilitated the manipulation of the surgical instruments within the constrained retroperitoneal space (Figure 1).

With the application of these techniques, the study periods were categorized into periods A, B, and C. Period A was the initial surgical approach before applying any new methods; period B was when LDF was applied on the soles of feet; and period C represented the time when LDF and PMR were used during ELS.

The primary outcome was the reappearance of plantar sweating after ELS. We did not consider this a recurrence of PPLH, as the degree of plantar sweating that patients experience after ELS was various and most of them did not cause disruptions in quality of life. In addition, compensatory hyperhidrosis was assessed via a survey during outpatient visits at 3–6 months post-surgery. The severity was evaluated using the well-established hyperhidrosis disease severity scale (HDSS).

### 2.4. Surgical Technique

For PMR, patients were placed in the supine position with a 12.5–15 cm thick, soft pillow under both knees, which significantly reduced tension along the psoas muscle. Following the application of general anesthesia and endotracheal intubation, patients were placed in the Trendelenburg position, and tilted 20–30° to the contralateral side of the surgical area; the surgery usually started on the left side. Access to the retroperitoneal space was achieved through the insertion of three trocars. A 15 mm incision, usually 8–10 cm from the umbilicus, was made in the middle of the lateral abdominal wall. Sequentially, the layers of the abdominal muscles were delineated using army and right-angle retractors to expose the external oblique, internal oblique, and transversalis muscle. The peritoneum was visualized, and right-angle retractors facilitated the visualization of the retroperitoneal fat. A 12 mm trocar (Spacemaker TM Pro, Covidien^®^, Dublin, Ireland) with a “space maker” function was introduced after identifying a low-resistance retroperitoneal space through manual palpation, thus allowing for peritoneal manipulation and expansion of the surgical field. Subsequently, two 5 mm trocars were inserted under finger palpation to maintain continuous carbon dioxide inflation during the procedure. An additional 5 mm trocar was introduced below the navel for de-airing in the event of peritoneal injury and inflation of intraperitoneal cavity.

The first step was to identify the psoas muscle and perform dissection along the surface to avoid injury to the ureter or lumbar vessels. For the right-sided approach, accurate identification of the lumbar sympathetic chain was ensured by locating the space between inferior vena cava and psoas muscle. LDF monitoring was used to improve the accuracy when visualizing the chain. A rapid drop and subsequent recovery of plantar blood flow were observed after clipping the right lumbar sympathetic chain, indicating that the entire chain was clipped. Electrocauterization using a Bovie hook was initially performed; however, the clipping technique was later adopted to prevent possible injury to the adjacent structures by cauterization. Antiadhesive agents were applied along the clipped chain, and the procedure was completed.

### 2.5. Statistical Analysis

For continuous variables (body mass index [BMI], height, weight, and operating time), the median and interquartile range were calculated after testing for normality by Shapiro–Wilk Test. The Mann–Whitney U test was used for non-normal continuous variable analysis. The chi-square test or Fisher’s exact test was used to analyze categorical variables. Risk factors for the reappearance of plantar sweating were identified using univariate and multivariate logistic regression analyses. To evaluate the effect of concomitant ETS on the development of compensatory hyperhidrosis, patients who underwent ETS before ELS were excluded. Logistic regression was also used to identify the relevant factors for compensatory hyperhidrosis among the 395 patients who underwent ELS ± ETS. Statistical analysis was conducted using the R package, version 4.2.2. Statistical significance was defined as *p <* 0.05.

## 3. Results

### 3.1. Patient Characteristics and Surgical Outcomes

**Figure 2:**
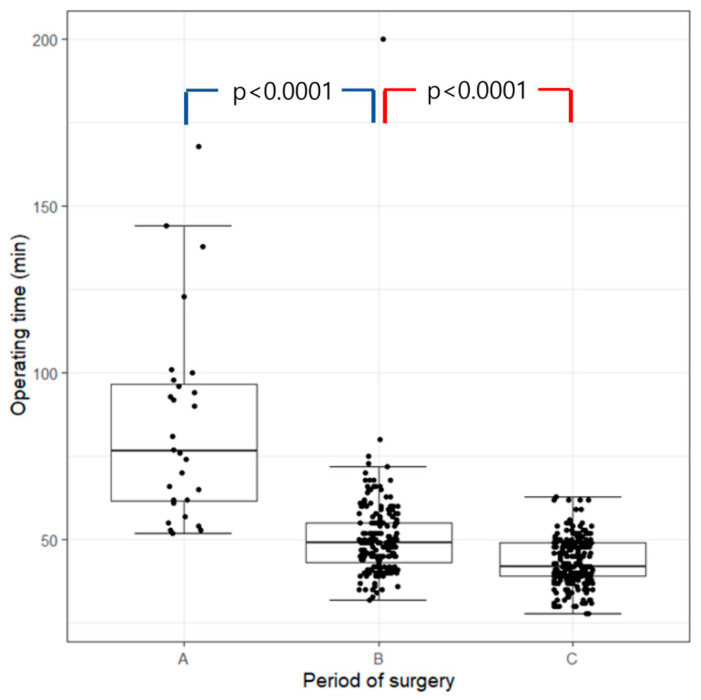
Variations in operation time across periods A, B, and C. Period A signifies the initial surgical approach before incorporating any new method; period B represents the phase when LDF is applied to both soles of the feet; and period C represents the timeframe when LDF is used on the soles of both feet and the PMR position is applied to patients during surgery. LDF, laser Doppler flowmetry; PMR, psoas muscle relaxation.

This study included 474 patients, and 277 (58.4%) were female (Table 1). A total of 79 (16.7%) patients underwent concurrent ETS. The patient cohort was divided into 28, 198, and 248 patients in periods A, B, and C, respectively. Though there was no significant statistical difference in terms of demographic in these periods, significant improvement was noted in operating time (Figure 2), as well as peritoneal injury (*p =* 0.001), and the reappearance of plantar sweating (*p =* 0.002).

### 3.2. Reappearance of Plantar Sweating After ELS and Risk Factor Analysis

Among the 474 patients, 21 (4.4%) reported the reappearance of plantar sweating during follow up. Within the reappearance group, eight patients (38.1%) had an unidentifiable lumbar sympathetic chain during surgery and seven patients (33.3%) had a history of lumbar sympathetic ganglion block (LSGB) prior to ELS. Moreover, the operation time was significantly longer in the recurrence group (Table 2). Based on a risk factor analysis, age >35 years [OR (95% CI): 4.57 (1.56–13.40), *p =* 0.006] and a history of LSGB [OR (95% CI): 269 (29.30–2460), *p <* 0.001] were identified as significant risk factors for reappearance (Table 3).

### 3.3. Compensatory Hyperhidrosis

To find risk factors related to compensatory hyperhidrosis, the study cohort was further narrowed to 395 patients after excluding 79 patients who had undergone ETS prior to ELS (SDC Table S1). Then, we compared two groups: 24 patients who underwent ELS alone, and 371 patients who underwent concomitant ETS and ELS. In the ELS alone group, 4 patients (16.7%) experienced compensatory hyperhidrosis, whereas in the ELS + ETS group, 202 patients (54.4%) exhibited it. The severity of compensatory hyperhidrosis in the ELS group was mostly minimal with HDSS grade 1, whereas in the ELS + ETS group, more patients (*n =* 8, 2.2%) exhibited high degrees of compensatory hyperhidrosis (SDC Table S1).

The sites of compensatory hyperhidrosis included the palmar regions in three patients and the inguinal area in one patient within the ELS group, whereas in the ETS + ELS group, various areas were affected, with the back (58.4%) being the most common area (SDC, Table 1). Risk factor analysis for compensatory hyperhidrosis revealed age >25 years [OR (95% CI): 0.60 (0.40–0.90), *p =* 0.014] and concomitant ETS [OR (95% CI): 5.63 (1.88–16.90), *p =* 0.002] as significant risk factors (SDC Table S2).

## 4. Discussion

This study demonstrated excellent clinical outcomes for ELS and analyzed the benefits of several techniques to improve the procedure. Of note, it firstly attempted to delineate risk factors related to poor clinical outcomes, such as the reappearance of plantar sweating and compensatory hyperhidrosis, after ELS. In addition, this study exhibited results from the largest ELS cohort to date with a high success rate (95.6%). Among patients who underwent ELS alone, there was no patient who experienced compensatory hyperhidrosis with HDSS grade 2 or more. Although our study showed a lower success rate than that reported by Rieger et al. (97%) and Lima et al. (98%), this discrepancy can be attributed to our stringent definition of surgical success and the inclusion of more patients over a shorter period [5,19]. As illustrated in this study, ELS demonstrated a success rate comparable to that of ETS and appeared advantageous in terms of long-term outcome.

Unlike ETS, ELS has several technical challenges due to various and complicated retroperitoneal space anatomies [18]. It is crucial to identify the lumbar sympathetic chain during surgery without injuring critical adjacent structures such as the inferior vena cava [8]. ELS requires meticulous dissection of loose tissues without damaging the peritoneum, unlike ETS, in which gas insufflation or one-lung ventilation can easily ensure the visibility of thoracic sympathetic chain [20]. Hence, even minor bleeding or anatomical variations in the lumbar sympathetic chain can pose significant challenges [21]. In our study, LDF aided in accurate identification of the lumbar sympathetic chain, and the PMR position enhanced the dissection of the psoas muscle and vertebral junction, increasing instrument manipulation and facilitating ease in the procedure [18]. Additionally, the absence of known complications, such as retrograde ejaculation, other sexual dysfunction, or neuralgia post-ELS, suggests that advances in surgical techniques played an important role by lowering complication rate [2,5,22,23]. The substantial reduction in the operating time and detailed explanations regarding surgical techniques could potentially assist other surgeons in performing this procedure.

The present study identified unique risk factors associated with ELS failure—specifically the reappearance of plantar sweating. In all seven patients who had a history of LSGB administration, locating the lumbar sympathetic chain during ELS was not possible. This is possibly due to severe adhesions of the lumbar sympathetic nerve to loose connective tissues around the psoas muscle from the prior alcohol injection and fibrosis during the previous LSGB administration [24]. In this situation, adhesiolysis was attempted on the left side; however, it was not possible on the right side because of the risk of injury to the inferior vena cava or ureter. Therefore, it is vital for the surgeon to be aware of high-risk patients for surgery such as prior history of LSGB administration. Furthermore, age >35 years was also identified as a risk factor. This may be attributed to age-related degenerative changes in the psoas muscle and surrounding tissues. Further validation would be needed.

Our findings align with those of Loureiro et al., who showed that the ELS-only method is associated with a significantly lower incidence and less-severe manifestation of compensatory hyperhidrosis compared to ETS [20]. Patients who underwent concomitant ETS showed a higher incidence of compensatory upper trunk hyperhidrosis, which was likely influenced by the effects of ETS. Risk factor analysis indicated that concomitant ETS and age > 25 years contributed significantly to compensatory hyperhidrosis. Although concomitant ETS increased compensatory hyperhidrosis, the severity was low grade and not substantial, suggesting that simultaneous ELS and ETS might not negatively affect clinical outcome. Interestingly, age > 25 years emerged as a protective factor against compensatory hyperhidrosis, which was potentially linked to the proactive reporting of discomfort in younger age groups.

The present study had some limitations. As this was a retrospective single-center study, our findings might be influenced by selection biases Additionally, as there is no objective method for reporting the degree of sweating, we relied on the subjective judgment of the patients. Another limitation was the relatively short follow-up period. Further research is necessary to address these limitations and to improve our understanding of ELS outcomes.

## 5. Conclusions

In conclusion, ELS can be considered as a highly effective treatment option for treating plantar hyperhidrosis. Following ELS, the reappearance of plantar hyperhidrosis was observed in patients aged 35 years and older, as well as in patients with a history of LSGB. Based on these findings, ELS with careful patient selection may improve clinical outcome of patients with plantar hyperhidrosis.

## Figures and Tables

**Table 1:** Demographics and surgical outcomes for patients who underwent ELS.

Factor	Group	Total	Period A (Initial Period)	Period B (with LDF)	Period C (with LDF + PMR)	*p*-Value
		N = 474	*n =* 28	*n =* 198	*n =* 248	
**Sex**						0.512
	F	277 (58.4)	18 (64.3)	110 (55.6)	149 (60.1)	
	M	197 (41.6)	10 (35.7)	88 (44.4)	99 (39.9)	
**BMI**		22.1 [20.0, 24.1]	20.7 [18.9, 22.6]	22.8 [20.6, 24.6]	22.0 [20.0, 24.0]	0.004
**Obesity (BMI over 25)**		86 (18.1)	1 (3.6)	40 (20.2)	45 (18.1)	0.102
**Weight**		60.8 [53.0, 70.0]	56.5 [50.4, 66.8]	62.7 [53.9, 72.0]	60.0 [52.1, 69.5]	0.022
**Height**		165.6 [160.0, 173.0]	163.7 [159.3, 172.5]	165.5 [160.6, 173.3]	166.1 [160.0, 173.0]	0.614
**previous ETS**		79 (16.7)	13 (46.4)	33 (16.7)	33 (13.3)	<0.001
**Previous LSGB**		8 (1.7)	2 (7.1)	2 (1.0)	4 (1.6)	0.061
**Operating time**		46.0 [40.0, 52.0]	76.5 [61.7, 96.5]	49.0 [43.2, 55.0]	42.0 [39.0, 49.0]	<0.001
**Concomitant ETS**		390 (82.3)	13 (46.4)	163 (82.3)	214 (86.3)	<0.001
**Unidentifiable lumbar chain**		8 (1.7)	1 (3.6)	3 (1.5)	4 (1.6)	0.725
**Peritoneal injury**		38 (8.0)	4 (14.3)	25 (12.6)	9 (3.6)	0.001
**Reappearance of plantar sweating**		21 (4.4)	5 (17.9)	6 (3.0)	10 (4.0)	0.002
**Compensatory Hyperhidrosis**						0.005
	N	256 (54.0)	23 (82.1)	98 (49.5)	135 (54.4)	
	Y	218 (46.0)	5 (17.9)	100 (50.5)	113 (45.6)	
**Compensation degree**	HDSS0	256 (54.0)	23 (82.1)	98 (49.5)	135 (54.4)	0.027
	HDSS1	210 (44.3)	5 (17.9)	97 (49.0)	108 (43.5)	
	HDSS2+	8 (1.7)	0 (0.0)	3 (1.5)	5 (2.0)	

BMI, body mass index; ETS, endoscopic thoracic sympathectomy; HDSS, Hyperhidrosis Disease Severity Scale; LDF, laser Doppler flowmetry; LSGB, lumbar sympathetic ganglion block; PMR, psoas muscle relaxation.

**Table 2:** Clinical outcomes based on the presence of reappearance in plantar sweating post-ELS.

Factor	Group	No Reappearance	Reappearance	*p*-Value
		N = 453	N = 21	
**Sex**				0.503
	F	263 (58.1)	14 (66.7)	
	M	190 (41.9)	7 (33.3)	
**BMI**		22.1 [20.1, 24.2]	20.0 [18.7, 23.0]	0.003
**Weight**		61.2 [53.5, 70.1]	53.0 [50.1, 60.8]	0.012
**Height**		165.5 [160.0, 173.0]	165.9 [161.8, 170.0]	0.838
**Previous ETS**		73 (16.1)	6 (28.6)	0.138
**Previous LSGB**		1 (0.2)	7 (33.3)	<0.001
**Concomitant ETS**		379 (83.7)	11 (52.4)	0.001
**Operation Time**		46.0 [40.0, 52.0]	52.0 [42.0, 66.0]	0.009
**Compensatory hyperhidrosis**	N	236 (52.1)	20 (95.2)	<0.001
	Y	217 (47.9)	1 (4.8)	
**Peritoneal injury**		34 (7.5)	4 (19.0)	0.078
**Unidentifiable lumbar chain**		0 (0.0)	8 (38.1)	<0.001
**Study period**				0.008
	A	23 (5.1)	5 (23.8)	
	B	192 (42.4)	6 (28.6)	
	C	238 (52.5)	10 (47.6)	

BMI, body mass index; ETS, endoscopic thoracic sympathectomy; LSGB, lumbar sympathetic ganglion block.

**Table 3:** Analysis of risk factors associated with reappearance of plantar hyperhidrosis.

Univariate Analysis			Multivariate Analysis	
Factor	OR (95% CI)	*p*-Value	OR (95% CI)	*p*-Value
**Age over 35**	3.67 (1.47–9.21)	0.006	4.57 (1.56–13.40)	0.006
**BMI over 25**	0.46 (0.11–2.02)	0.314		
**Sex (Ref. Female)**	0.69 (0.27–1.75)	0.442		
**Previous ETS**	2.08 (0.78–5.54)	0.143		
**Previous LSGB**	226 (26.0–1960)	<0.001	269 (29.3–2460)	<0.001
**Intraoperative** **peritoneal injury**	2.90 (0.92–9.10)	0.068		

BMI, body mass index; ETS, endoscopic thoracic sympathectomy; LSGB, lumbar sympathetic ganglion block.

## Data Availability

Data is available upon reasonable request to the corresponding author.

## Supplementary Materials

The following supporting information can be downloaded at https://www.mdpi.com/article/10.3390/jcm14124311/s1: Table S1: Compensatory hyperhidrosis in relation to concomitant endoscopic thoracic sympathectomy; Table S2: Risk factor analysis for Compensatory hyperhidrosis.
