# Supplementary material for: Advances in Clinical Outcomes of Endoscopic Lumbar Sympathectomy: Analysis of 494 Consecutive Patients at a Single Institution
Source: J Clin Med. 2025 Jun 17;14(12):4311. doi: 10.3390/jcm14124311 (PMC12194193; doi:10.3390/jcm14124311)
Supplement: Supplementary file 1 [file jcm-14-04311-s001.zip › jcm-3672452-supplementary.pdf]

Supplementary Table S1. Compensatory hyperhidrosis in relation to concomitant endoscopic thoracic sympathectomy

Factor		ELS only, N=24	ELS + ETS N=371	p-value
**Compensatory Hyperhidrosis**				<0.001
	No	20 (83.3)	169 (45.6)	
	Yes	4 (16.7)	202 (54.4)	
**Degree of Compensatory hyperhidrosis**				0.002
	HDSS0	20 (83.3)	169 (45.6)	
	HDSS1	4 (16.7)	194 (52.3)	
	HDSS2	0 (0.0)	8 (2.2)	
**Site of compensatory hyperhidrosis**				<0.001
	abdomen	0 (0.0)	14 (7.4)	
	back	0 (0.0)	111 (58.4)	
	chest	0 (0.0)	12 (6.3)	
	face	0 (0.0)	7 (3.7)	
	flank	0 (0.0)	4 (2.1)	
	hip	0 (0.0)	2 (1.1)	
	inguinal	1 (25.0)	1 (0.5)	
	knee	0 (0.0)	3 (1.6)	
	leg	0 (0.0)	1 (0.5)	
	palmar	3 (75.0)	0 (0.0)	
	popliteal	0 (0.0)	2 (1.1)	
	thigh	0 (0.0)	33 (17.4)	

ELS, endoscopic lumbar sympathectomy; ETS, endoscopic thoracic sympathectomy; HDSS, Hyperhidrosis Disease Severity Scale

Supplementary Table S2. Risk factor analysis for Compensatory hyperhidrosis

	Univariate analysis		Multivariate analysis	
Factor	OR (95% CI)	p-value	OR (95% CI)	p-value
Age over 25	0.58 (0.39-0.86)	0.007	0.60 (0.40-0.90)	0.014
BMI over 25	0.93 (0.56-1.53)	0.770		
Male (Ref. Female)	0.90 (0.60-1.34)	0.590		
Concomitant ETS	5.98 (2.00-17.8)	0.001	5.63 (1.88-16.9)	0.002
Study period (Ref. period A)¶				
Period B	2.58 (0.85-7.89)	0.106		
Period C	2.02 (0.67-6.10)	0.210		
Intraoperative peritoneal injury	1.05 (0.50-2.22)	0.890		

BMI, body-mass index; ETS, endoscopic thoracic sympathectomy

Study periods were divided according to the introduction of laser doppler flowmetry (Period B) and intraoperative psoas muscle relaxation (Period C)
